# Suppression of SnS_2_ Secondary Phase on Cu_2_ZnSnS_4_ Solar Cells Using Multi-Metallic Stacked Nanolayers

**DOI:** 10.3390/nano13030432

**Published:** 2023-01-20

**Authors:** Fang-I Lai, Jui-Fu Yang, Jia-En Li, Yu-Chao Hsu, Shou-Yi Kuo

**Affiliations:** 1 Department of Electrical Engineering Program C, Yuan-Ze University, 135 Yuan-Tung Road, Chung-Li 32003, Taiwan; 2 Department of Urology, Chang Gung Memorial Hospital, Linkou, No. 5, Fuxing Street, Kwei-Shan, Taoyuan 333, Taiwan; 3 School of Medicine, Chang Gung University, 259 Wen-Hwa 1st Road, Kwei-Shan, Taoyuan 333, Taiwan; 4 Department of Electronic Engineering, Chang Gung University, 259 Wen-Hwa 1st Road, Kwei-Shan, Taoyuan 333, Taiwan

**Keywords:** bifacial solar cell, CZTS, nano multi-metallic stacked

## Abstract

In Cu_2_ZnSnS_4_ (CZTS) solar cells, it is crucial to suppress the generation of and remove the SnS_2_ secondary phase to improve the solar cell characteristics, as the SnS_2_ secondary phase affects the barrier for carrier collection and diode characteristics of the device. In this study, the nano-metallic precursor was modified to effectively suppress the generation of the SnS_2_ secondary phase on the surface and simultaneously improve the uniformity and quality of the thin film. The CZTS bifacial solar cells prepared via the proposed method exhibited significantly improved junction-rectifying characteristics, as the efficiency was improved to 1.59%. The proposed method to figurremove SnS_2_ is effective, simple, and environmentally friendly.

## 1. Introduction

With a rapidly increasing demand for energy, solar energy, among the various renewable energy sources, has attracted considerable attention. Commercially available silicon-based solar cells have limited efficiency gains, high costs, and terrain constraints, which hinder their development. Thin-film solar cells based on the second-generation solar cell technology mainly use different types of compounds in their absorber layer. Among them, Cu(In,Ga)Se_2_ (CIGS) and CdTe have already reached similar conversion efficiencies to those of silicon-based solar cells, and thus have attracted considerable attention [1,2,3]. However, considering the availability, toxicity, and costs of the compounds, they are not suitable for a sustainable development of the industrial chain. Therefore, it is crucial to develop an absorber layer using a compound that is universal, low-cost, nontoxic, and environmentally friendly, and that can be integrated into the existing production lines for CIGS and CdTe. Among the possible candidates, Cu_2_ZnSnS_4_ (CZTS) has attracted considerable attention. CZTS is a semiconductor material with a direct band gap (E_g_ = 1–1.5 eV) and large light absorption coefficient (>10^4^ cm^−1^) in the visible spectrum [4]. With a thickness of only 1–2 µm, it can absorb most of the sunlight, effectively saving space while providing better flexible characteristics. Thus, it is of interest for future applications. The preparation processes of the thin-film absorber layer for the CZTS solar cells can be categorized into vacuum and nonvacuum processes. Common vacuum processes include sputtering and deposition [5,6], while nonvacuum processes include electroplating, the sol–gel process, spray coating, and spin coating [7,8,9]. The highest efficiency of CZTS solar cells has been 12.2% [10]. Molybdenum (Mo) is still the most common back-contact material for kesterite devices, due to the interaction with the chalcogens during the absorber formation process at an elevated temperature where a MoS_2_ layer is formed at the Mo/kesterite interface. The formation of Mo/MoS_2_/CZTS junctions may exhibit features such as ohmic contact, potential barriers, or form reverse (*n*-p) diodes. Under the proper thickness control of MoS_2_, an ultrathin MoS_2_ layer is known to improve contact ohmicity and is often deemed to be necessary in high-efficiency devices. So far, state-of-the-art CZTS−based photovoltaic devices are almost fabricated on Mo substrates [11,12,13]. However, in some applications that require a transparent back contact, such as bifacial or tandem solar cells, Mo must be replaced by another material to achieve substrate transparency and conductivity. Despite the intense research effort on alternative materials, conventional TCOs remain the most common transparent conductors. Kesterite solar cells using FTO and ITO as transparent back contacts have been reported. Mali et al. were the first to report the device efficiency of 1.9% after the deposition of CZTS on FTO using the SILAR method [14]. Mahajan et al. used a similar approach to deposit CZTS on both ITO and FTO, also reaching similar device results of 1.68% and 2.08%, respectively [15]. Ge et al. demonstrated that the performance of CZTS bifacial solar cells via co-electroplating with ITO back contact exhibits an efficiency of 3.4% [16]. Without any additional interfacial layers, the possibility of directly employing FTO or ITO substrates for the fabrication of efficient CZTS photovoltaic devices has already been demonstrated with efficiencies of up to 4.73% and 5.8% [17,18]. These efficiency values are well below those of the champion kesterite devices fabricated on standard Mo substrates. One of the main reasons may be ascribed to the non-ohmic contact and complex behavior of the kesterite/TCO back interface that may hinder charge extraction, though different strategies can be employed to reduce or even prevent these problems. Taking the chemical and thermal stability into consideration, fluorine-doped tin oxide seems to be a potential candidate for kesterite-based devices.

According to the Shockley–Queisser limit, the theoretical limit of the efficiency of CZTS solar cells is above 30%. The difference between the achieved efficiency and the theoretical limit may be caused by the growth parameters of the CZTS thin film, such as vulcanization parameters [19,20] and the stacking order of the precursor layers [21,22]. These parameters affect the composition and quality of the CZTS absorber layer; e.g., they cause the formation of secondary phases in the absorber layer [23,24]. In particular, Sn-based binary compounds are not only harmful to the device, but are also difficult to control, because Sn-based binary compounds are highly volatile, which can easily reevaporate from the absorber layer at a high temperature, resulting in changes in the composition and defect states in the absorber layer. Sn-based deep defects may be formed, leading to the formation of recombination centers, which affect the band gap of the absorber layer. Moreover, the reevaporated SnS_x_ is easily adsorbed on the surface of the thin film, affecting the collection of carriers, thus deteriorating the characteristics of the device. To suppress the formation of SnS_x_ on the surface, in 2014, Xie et al. discovered that a (NH_4_)_2_S chemical solution could successfully remove the Sn(S,Se) secondary phase on the surface of the CZTSSe absorber layer. In addition, the surface is passivated to reduce the surface recombination, such that, compared to the unetched device, the etched device exhibited an efficiency improvement of up to approximately 65% [25]. In 2017, Wang et al. reported that SnS_x_ was formed through condensation on the surface of the CZTS thin film during the natural cooling process after high-temperature vulcanization. The SnS_2_ secondary phase on the surface of the CZTS thin film could be peeled off through physical adhesion using a conductive tape that does not easily debond. The device with removed SnS_x_ exhibited an increase in efficiency from 0.38% to 4.3% [26]. Although the above methods can effectively remove SnS_x_ on the surface, the Sn-based defects inside the absorber layer may not be effectively removed. Therefore, in this study, we investigate the suppression of Sn-based defects through the optimization of the reaction mechanism of the precursor, to improve the quality of the thin-film absorber layer in a simple, convenient, and environmentally friendly manner.

## 2. Materials and Methods

**Figure 1:**
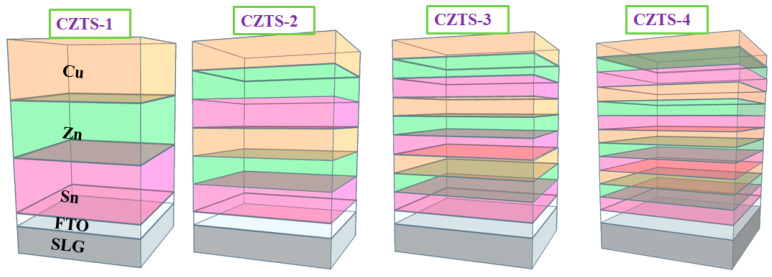
Schematic of precursor stacks.

The CZTS thin films were prepared via deposition vulcanization. First, Cu/Sn/Zn nano-metallic precursors (each of one layer) were sequentially deposited on a commercially available fluorine-doped tin oxide (FTO)(SnO_2_:F) glass substrate. By dividing the Cu/Sn/Zn precursors, precursors of two, three, and four layers (with an unchanged total thickness of each metal, as shown in Figure 1) were then prepared. The metal used for vapor deposition had a purity of 99.999% (purchased from Summit-Tech Resource Corp., Hsinchu, Taiwan) and was a commercial FTO with a sheet resistance of approximately 20 Ω/□ (Ruilong Optoelectronics Co., ltd., Miaoli, Taiwan). Second, the four groups of deposited precursors were placed into a quartz box (with an excellent air tightness). Sulfur powder in the quantity of 6000 mg was also added into the quartz tube for vulcanization. During vulcanization, the highest temperature was 500 °C, and Ar was introduced continuously. The reacted gas was filtered out with a carrier using the suction component of a dry adsorption tower. The vulcanized specimens are denoted as CZTS−1 (with one layer), CZTS−2 (with two layers), CZTS−3 (with three layers), and CZTS−4 (with four layers) according to the number of precursor stacks. The microstructure, morphology, and optical properties of the thin films were then measured using X-ray diffraction (XRD), field-emission scanning electron microscopy (FE-SEM), energy-dispersive spectroscopy (EDS), Raman scattering, ultraviolet (UV)–visible (Vis)–near-infrared (NIR) spectrophotometry, micro-Raman spectroscopy, and secondary-ion mass spectroscopy (SIMS) to analyze the changes in the qualities of the thin films. Subsequently, chemical bath deposition was carried out to prepare a layer of CdS with a thickness of approximately 50 nm, in which the CdS buffer layers were grown via chemical bath deposition using Cd(CH_3_ CO_2_)_2_ 2H_2_O and CH_4_N_2_S solutions, while sputtering was carried out to prepare an i-ZnO (99.95% purity) layer with a thickness of approximately 50 nm and AZOY (99.95% purity) layer with a thickness of approximately 300 nm. Afterward, sputtering was again carried out to deposit a Ni layer with a thickness of approximately 50 nm and an Al layer with a thickness of approximately 1 µm as an upper electrode (with an effective area of the CZTS device of 0.18 cm^2^). All target materials had a dimension of three inches, and were purchased from Summit-Tech Resource Corp. The photoelectric characteristics of the prepared CZTS solar cell were evaluated using a current density–voltage (J–V) measurement at ambient temperature controlled at 25 ± 1 °C. Illumination was performed using a solar simulator that generates the AM 1.5 global spectrum.

## 3. Results and Discussion

**Figure 2:**
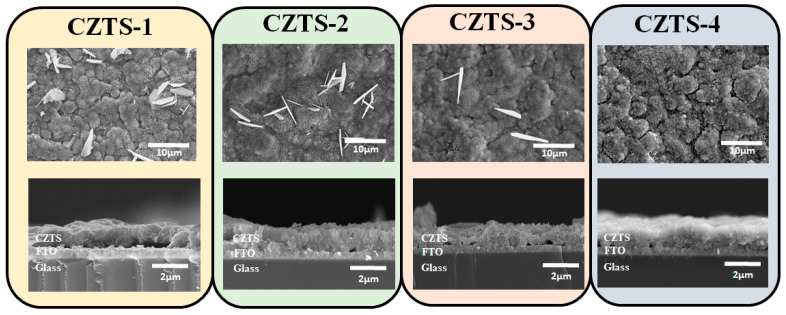
SEM top and cross-sectional views of CZTS−1, CZTS−2, CZTS−3, and CZTS−4.

Figure 2 shows SEM top and cross-sectional views of CZTS−1, CZTS−2, CZTS−3, and CZTS−4. The cross-section atomic percentage was measured using EDS. The results are shown in Table 1. Although the number of precursor stacks increases, the relative changes in the atomic percentages of Cu, Zn, and S atoms are not large: 0.7%, 0.97%, and 1.4%, respectively. The relative change in the atomic percentage of Sn atoms is largest. The cause of the change in atomic percentage is likely the change in the number of corresponding nano-metallic precursor stacks. When the number of Cu–Sn junctions increases, Cu_6_Sn_5_ alloys can form more easily due to the high diffusivity of the Cu atoms [27]. Thus, the increase in the number of nano-metallic precursor stacks leads to an increase in the content of the Cu6Sn5 alloy distributed inside the precursor. This can suppress the loss of Sn atoms during the vulcanization process at a high temperature, thus increasing the atomic percentage of Sn. In addition, Figure 2 shows that, while all CZTS thin films have a thickness of approximately 1.2 μm, sheet-like objects were formed on the surfaces of CZTS−1, CZTS−2, and CZTS−3, which are supposed to be SnS_x_ compounds according to the literature. The presence of SnS_x_ on the surface of CZTS thin films severely affects the device, which reduces open circuit voltage (V_oc_) of the devices by forming a diode and a barrier for carrier collection [26]. Moreover, as the number of precursor stacks increases, the amount of SnS_x_ gradually decreases, so that no obvious sheet-like objects were formed on the surface of the CZTS−4 specimen. This may be attributed to the relatively small amount of Cu_6_Sn_5_ alloy formed inside the thin film at a high temperature when the number of precursor stacks is small, which corresponds to a higher loss of SnS_x_. The evaporated SnS_x_ cannot escape the quartz box due to the air tightness, but remains inside the quartz box and becomes adsorbed to the surface under natural cooling as it is difficult for SnS_x_ to react with Zn on the surface of the specimen to form a different compound.

**Figure 3:**
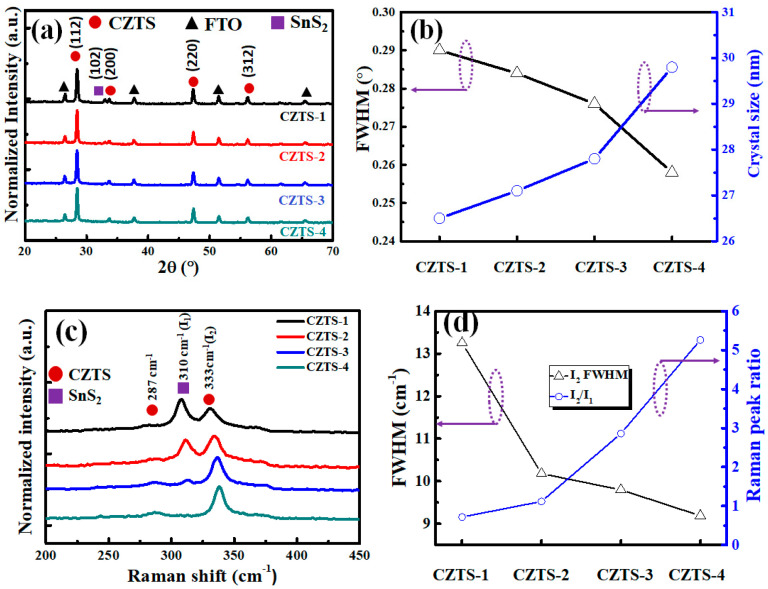
(**a**) XRD measurements, (**b**) FWHM and crystal size as a function of the number of precursor stacks for the (112) plane obtained by the XRD measurements, (**c**) Raman spectra, and (**d**) Raman [I_2_] FWHM values, and Raman [I_2_]/[I_1_] band intensity ratio as a function of the number of precursor stacks of CZTS−1, CZTS−2, CZTS−3, and CZTS−4.

**Figure 4:**
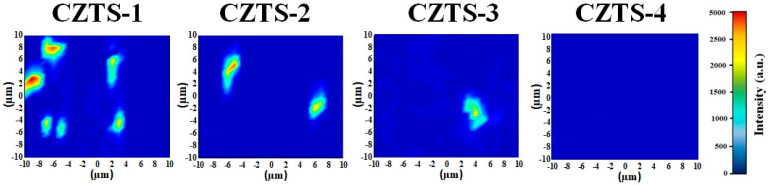
Raman mapping of CZTS−1, CZTS−2, CZTS−3, and CZTS−4.

Figure 3a shows XRD measurement results of CZTS−CZTS−1, CZTS−CZTS−2, CZTS−CZTS−3, and CZTS−4 (plotted with normalized data). All specimens exhibited diffraction peaks corresponding to (112), (200), (220), and (312) planes of CZTS and FTO substrate. The measurements of CZTS−1, CZTS−2, and CZTS−3 show a diffraction peak corresponding to the (102) plane of the SnS_2_ secondary phase, whose intensity decreases as the number of precursor stacks decreases. In contrast, there was no obvious SnS_2_ diffraction peak for CZTS−4. The XRD measurements were consistent with the SEM measurements. As the grain size of the CZTS thin film can be evaluated using the Scherrer equation, the full width at half maximum (FWHM) calculated based on the CZTS (112) plane was used to evaluate the grain size and quality of the CZTS thin film [28]:

(1) $$D=\frac{0.9\lambda }{\beta \cos\theta \prime }$$

where D is the grain size, *λ* is the wavelength of the X-rays (0.154 nm), β is the FWHM of CZTS (112) measured by XRD, and θ is the Bragg angle. Figure 3b shows the FWHM and crystal sizes calculated based on the CZTS (112) plane. The quality of the vulcanized thin film improves as the grain size increases, likely because the atoms in the precursor are more easily and uniformly diffused as the number of precursor stacks increases. Moreover, the content of the Cu_6_Sn_5_ alloy formed in the precursor increases, which is conducive to crystal growth [29]. As XRD is a macroscopic measurement method, while CZTS has diffraction peaks that are close to those of many secondary phases, and thus the diffraction peaks are difficult to distinguish, Raman spectroscopy was used to further understand the formation of relevant compounds. Figure 3c shows the Raman spectra of CZTS−1, CZTS−2, CZTS−3, and CZTS−4 under the A1 mode (after the data were normalized). Overall, considerable signals were captured at approximately 287 and 333 cm^−1^, which correspond to CZTS [30,31], while the signal at approximately 310 cm^−1^ corresponds to SnS_2_ [32]. To further quantify the ratio of SnS_2_ to CZTS inside the absorber layer, the signal intensity at 310 cm^−1^ was defined as I_1_, while the signal intensity at 333 cm^−1^ was defined as I_2_, such that I_2_/I_1_ represents the ratio of SnS_2_ to the CZTS thin film. The FWHM values calculated based on the I_2_ signal are shown in Figure 3d. Figure 4 shows that FWHM is largest for CZTS−1, followed by those of CZTS−2, CZTS−3, and CZTS−4. I_2_/I_1_ also obeys the order of CZTS−1, CZTS−2, CZTS−3, and CZTS−4. This indicates that, as the number of precursor stacks increases, both the quality of the CZTS thin film and the ratio of SnS_2_ on the surface of the thin film change consistently with the previous results.

To confirm whether the sheet-like objects in the SEM images are SnS_x_ phase, and to facilitate the understanding of how the distribution of the SnS_x_ phase changes on the surface of the absorber layer, Raman mapping was carried out to measure the surface of each specimen. Figure 4 shows Raman mapping images of CZTS−1, CZTS−2, CZTS−3, and CZTS−4. Under the A1 mode, signals with a frequency between 310 and 313 cm^−1^ correspond to the SnS_x_ secondary phase. The planar distribution of the composition of the SnS_x_ secondary phase is shown, with a measurement area of 20 × 20 µm^2^. As the number of precursor stacks increases, the amount of SnS_x_ on the surfaces of CZTS−1, CZTS−2, and CZTS−3 exhibits a decreasing trend. In the case of CZTS−4, no SnS_2_ was observed. The Raman mapping analysis shows that the SnS_x_ secondary phase content on the surface of the specimen decreases as the number of precursor stacks increases, which is consistent with the measured XRD and SEM results.

**Figure 5:**
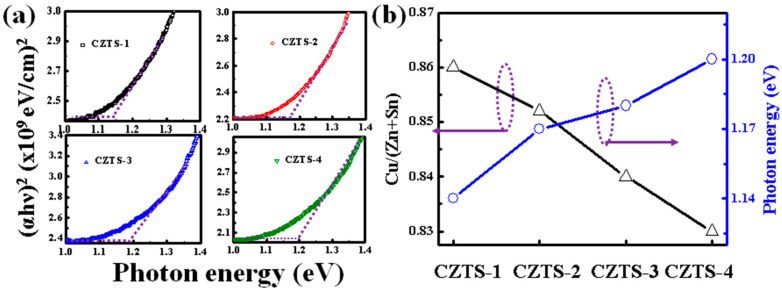
(**a**) Band gap, (**b**) Cu/(Zn + Sn) ratio, and band gap as a function of the number of precursor stacks of CZTS−1, CZTS−2, CZTS−3, and CZTS−4.

Figure 5a shows the transmittance and reflectance (not shown here) measured for CZTS−1, CZTS−2, CZTS−3, and CZTS−4 deposited on the FTO substrate using UV–Vis–NIR spectra. The following equation was used to calculate the absorption coefficient [33]:

(2) $$\alpha =\frac{1}{t}ln\left[\frac{{\left(1-R\right)}^{2}}{T}\right],$$

where *t* is the thickness of the CZTS thin film. For a certain type of optical transition, the relationship between the optical band energy and absorption coefficient can be expressed as

(3) $${\left(\alpha h\upsilon \right)}^{2}=A\left(hv-E_{g}\right),$$

where *α* is the absorption coefficient, *h* is Planck’s constant, *v* is the photon frequency, and *A* is a constant. According to the plot of (*ahv*)^2^ against the photon energy (*hv*), (*ahv*)^2^ is linearly extrapolated to calculate the band gap considering the intercept on the axis of *hv* [33]. To further understand the reason for the changes in band gap, the number of precursor stacks, the band gap, and the ratio of Cu/(Zn + Sn) are plotted in Figure 5b. As the number of precursor stacks increases, the ratio of Cu/(Zn + Sn) gradually decreases, while the band gap gradually increases. This may be attributed to the new hybridization orbitals formed by the d orbitals of the Cu atoms and p orbitals of the S atoms, which are consistent with the changes in the literature [34].

**Figure 6:**
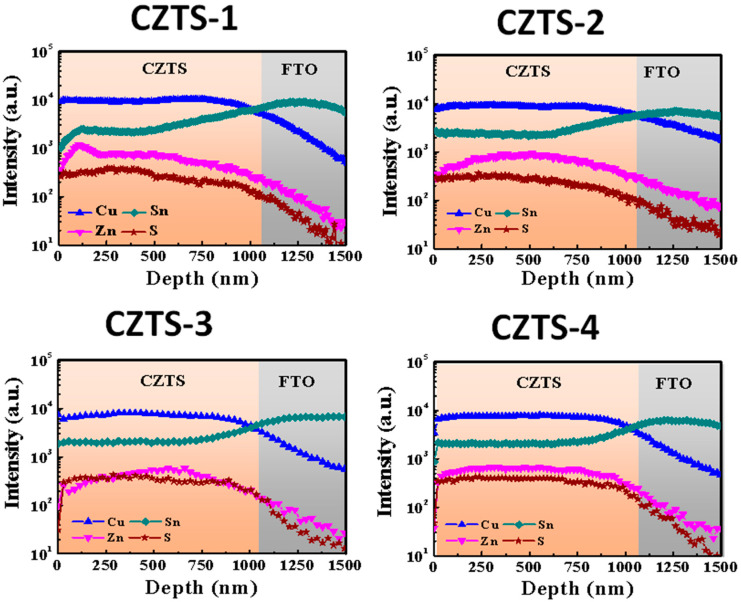
SIMS measurement results of CZTS−1, CZTS−2, CZTS−3, and CZTS−4.

To further understand the distribution of each element inside the CZTS thin film, SIMS measurements were carried out for CZTS−1, CZTS−2, CZTS−3, and CZTS−4, as shown in Figure 6. Each figure contains two parts, showing the measurements for CZTS and FTO. The elemental distribution in the CZTS−1 thin film absorber layer was not uniform. However, as the number of precursor stacks increases, the elemental distribution inside the CZTS thin films becomes more uniform.

**Figure 7:**
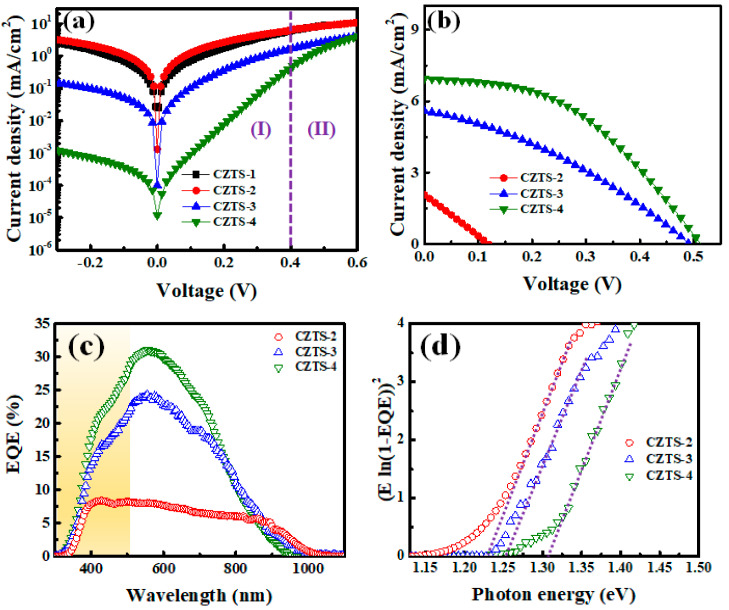
J–V measurements (**a**) in the dark and (**b**) under AM 1.5G light for CZTS−1, CZTS−2, CZTS−3, and CZTS−4. (**c**) EQE of CZTS−2, CZTS−3, and CZTS−4 samples under AM1.5 illumination; (**d**) band gap determination of the cell from the EQE data.

Figure 7a,b shows J–V plots for the devices fabricated using CZTS−1, CZTS−2, CZTS−3, and CZTS−4 using different environmental parameters in the dark and under AM1.5G light, respectively. The characteristics of the devices are listed in Table 2.

The measurement results in the dark can be divided into two different regions. Region I covers the measurement range between −0.3 and 0.4 V, where the current density is affected mainly by the shunt leakage current and space charge. Region II covers the measurement range between 0.4 and 0.6 V, where a diode current is observed. Region I shows that the shunt leakage current decreases as the number of precursor stacks increases, which indicates that the leakage current is affected by the amount of SnS_x_ on the CZTS surface. As the nano-metallic precursor in CZTS−1 is thick and not conducive to diffusion, the elements cannot be distributed uniformly in the thin film. In contrast, with the modified nano-metallic precursor, the diffusion of the precursor between the nano-metallic layers is facilitated, and thus the elemental distribution within the thin film becomes more uniform. In addition, as the Cu_6_Sn_5_ alloy content increases, the loss of Sn is reduced, which reduces the amount of SnS_x_ on the surface of the CZTS thin film. For the CZTS photovoltaics devices, the characteristics of series resistance (R_s_), parallel resistance (R_sh_), and ideality factor (*n*) are also important. Series resistances are mainly caused by bulk materials and metal contact, while shunt resistances are mainly caused by defects within the bulk materials and interfaces. A high R_sh_ value usually indicates a low leakage current. The calculated values of R_s_, R_sh_, and *n* for the samples CZTS−1 to CZTS−4 are listed in Table 2, which shows that as the number of stacks increases, R_sh_ gradually increases. This is due to the reduction in the amount of SnS_2_ on the surface of the absorber layer, which reduces the leakage path. In contrast, R_s_ decreases as the number of stacks increases, presumably owing to the decrease in the amount of secondary phases on the surface. The ideality factor can be used to determine the primary carrier transport mechanism of the heterojunction. When *n* = 1, the primary carrier transport mechanism is mainly diffusion; when *n* > 2, the carrier transport mechanism is mainly recombination and generation in the depletion region; and when *n* > 6, the carrier transport mechanism is dominated by the defect states at the interface [35]. From Table 2, it can be seen that the ideality factors of the specimens CZTS−1 and CZTS−2 are greater than six, and carrier transport is mainly affected by the interface states and decreases as the number of stacked metallic layers increases. For CZTS−3 and CZTS−4, carrier transport is dominated by recombination and generation in the depletion region. This also suggests the presence of interface states and defects in the space-charge region, which act as charge carrier traps. The junction-rectifying characteristics of the device can be expressed by the ratio between the currents at 0.3 and −0.3 V measured in the dark (i.e., I_dark_ [0.3 V]/I_dark_ [−0.3 V]). A larger junction-rectifying characteristic implies a better junction performance [36]. The junction-rectifying characteristics of the devices with CZTS−1, CZTS−2, CZTS−3, and CZTS−4 are listed in Table 2. As the number of precursor stacks increases, the junction-rectifying characteristics gradually improve. The best junction-rectifying characteristics are obtained for CZTS−4. Under AM 1.5G light, as the number of precursor stacks increases, the photoelectric efficiency, open-circuit voltage, short-circuit current, and fill factor gradually improve, mainly because the SnS_x_ secondary phase on the CZTS surface gradually decreases, which improves the diode devices. External quantum efficiency (EQE) measurements are helpful in understanding the carrier loss mechanism of CZTS devices. Figure 7c shows the EQE response of solar cells from CZTS−2 to CZTS−4. It can be seen that a significant EQE loss occurs below the wavelength of ~510 nm (yellow part), which is mainly due to the material absorption of the CdS and ZnO layers. The EQE loss in the range of wavelengths greater than 510 nm is mainly affected by the amount of the SnS_x_ secondary phase. A high amount of SnS_x_ will increase the defect density, which increases the recombination rate in solar cells. As the number of precursor stacks increases, the EQE loss decreases significantly, which is consistent with the improvement in the junction-rectifying characteristics of the devices. In addition, according to the EQE curves, the energy gap (E_g_) value can be calculated by a linear extrapolation (E × ln(1-EQE))^2^ and E (as shown in Figure 7d). The energy gaps of the CZTS−2, CZTS−3, and CZTS−4 devices were estimated to be 1.23 eV, 1.25 eV, and 1.3 eV, respectively, and their E_g_ trends were consistent with the UV–Vis–NIR measurement results. Therefore, from the J–V and EQE analyses, it was found that the presence of sheet-like SnS_x_ on the CZTS surface greatly reduces the conversion efficiency of CZTS photovoltaic devices, while increasing the number of precursor stacks would effectively reduce the loss related to the SnSx secondary phase.

## 4. Conclusions

As the number of precursor stacks increases, the SnS_2_ secondary phase on the surface of the CZTS thin film can be effectively suppressed, while the uniformity of the elemental distribution as well as the crystal size of the thin film are improved. This may be related to the increase in the content of the Cu_6_Sn_5_ alloy attributed to the increase in the number of precursor stacks, which not only suppresses the loss of Sn at a high temperature during the vulcanization process and reduces the nonuniformity of the elemental distribution in the absorber layer, but also facilitates the crystal growth in CZTS. In this study, a simple, nontoxic, and environmentally friendly approach was used to improve the quality of the CZTS thin film and remove the SnS_x_ secondary phase on the surface, which is valuable for the development of highly efficient solar cells.

## Figures and Tables

**Table 1:** Atomic percentages of CZTS−1, CZTS−2, CZTS−3, and CZTS−4.

Sample	Cu	Zn	Sn	S
**1**	22.18	14.46	11.46	51.90
**2**	22.21	14.35	11.73	51.71
**3**	22.28	14.34	11.84	51.54
**4**	22.35	14.32	12.16	51.17

**Table 2:** Device characteristics of CZTS−1, CZTS−2, CZTS−3, and CZTS−4.

Sample	V_oc_ (V)	J_sc_ (mA/cm^2^)	FF (%)	Efficiency (%)	Junction Rectifying Characters	*n*	R_sh_ (KΩ˙cm^2^)	R_s_ (Ω˙cm^2^)
**CZTS−1**	Not applicable	Not applicable	Not applicable	Not applicable	1.3	9.65	0.09	560
**CZTS−2**	0.12	2.07	24.15	0.06	1.35	6.46	0.1	400
**CZTS−3**	0.49	5.6	33.89	0.93	6.22	5.34	1.17	310
**CZTS−4**	0.51	7.15	43.6	1.59	45.84	2.3	10.68	34

## Data Availability

The data presented in this study are available upon request from the corresponding author.
